# Relationship Between Dispositional Mindfulness and Living Condition and the Well-**B**eing of First-**Y**ear University Students in Japan

**DOI:** 10.3389/fpsyg.2019.02831

**Published:** 2019-12-18

**Authors:** Tomonari Irie, Kengo Yokomitsu

**Affiliations:** ^1^School of Education and Culture, Hokusho University, Hokkaido, Japan; ^2^College of Comprehensive Psychology, Ritsumeikan University, Osaka, Japan

**Keywords:** first-year university students, mental health, well-being, dispositional mindfulness, living condition

## Abstract

The present study was conducted to examine how dispositional mindfulness and living conditions are related to well-being among first-year university students in Japan. Participants were 262 Japanese first-year students (156 females and 106 males; *M*_age_ = 18.77 years, *SD*_age_ = 0.85). Dispositional mindfulness was measured using the Mindful Attention Awareness Scale (MAAS), and living condition was operationalized as living at home or living alone after having left their home. Hierarchical multivariate regression analysis was used to analyze whether the factors of living condition and dispositional mindfulness had predictive effects on well-being. The results showed that dispositional mindfulness positively correlated with well-being in first-year university students; however, living condition had no significant correlation. On the other hand, the interaction between living condition and dispositional mindfulness significantly correlated with well-being. Simple slope analysis revealed that higher levels of dispositional mindfulness had a protective effect in the relationship between living condition and well-being. These results suggest that an intervention to promote dispositional mindfulness could be effective in protecting the well-being of first-year university students, especially for those who have left their home and are living alone. Further research will be necessary to examine, longitudinally, how mental health changes depending on the level of dispositional mindfulness of first-year university students.

## Introduction

The American College Health Association (2015) reported that approximately half of American university students experience mental health problems. University students who have psychiatric disorders are reluctant to attend university and are often unable to seek appropriate treatment (Auerbach et al., 2016). Additionally, mental health problems have been found to have a negative impact on academic performance (Riglin et al., 2014). This trend occurs not only in Western countries such as the United States, England, and Germany, but also in Asian countries such as Japan, South Korea, and Thailand (Steptoe et al., 2007). Due to these concerns, mental health problems is especially important among first-year university students. Research has shown that first-year university students report more mental health problems than students from other years (Bewick et al., 2010), which may partly be due to them living separately from their families. Many of these first-year students are living on their own and need to take care of themselves for the first time in their lives (Sallis et al., 2008). Moreover, it has been pointed out that when adolescents live away from their parental home, they may experience poor social connections, loneliness, and difficulty in obtaining social support (Wannebo et al., 2018; Dvořáková et al., 2019). This means that living on their own may have a negative impact on their mental health, compared to living at their parental home. Transitioning to university and living on their own forces students to establish new social support systems (Rodgers and Tennison, 2009). As a result, these students may have difficulty adapting to university life and may experience mental health problems (Dyson and Renk, 2006). The transition to university has been said to be an acute stressor, having a strong negative impact on mental health (Gall et al., 2000). Consistent with prior research in other countries, first-year Japanese university students report more mental health problems than students in other years (Irie et al., 2015). First-year university students with poor mental health also have more problems with academic activities and social relationships during their second year (Watanabe and Watanabe, 2015). First-year university students with poor well-being are also more likely to take temporary leave or to drop-out from university (Murai and Nakayama, 2008); a study using a survival analysis has also shown that poor mental health in the first year of university increases the risk of dropping out (Irie and Maruoka, 2017). This, in turn, may affect students’ future career prospects. For example, only 1.7% of people who drop out of university work as full-time permanent employees for the next three consecutive years. In comparison, 60% of university and graduate school graduates are working as permanent employees (The Japan Institute for Labour Policy and Training, 2015). Therefore, university students’ mental well-being is not merely a health care issue; it also has cumulative effects on the socioeconomic welfare of a society.

The transition from high school to university is generally considered to be a positive step in personal development, as it is an important life challenge to stand on one’s own two feet (Bernier et al., 2005). First-year university students with greater well-being show better academic performance (Baumann et al., 2014). Mental health is defined as a “state of well-being in which every individual realizes his or her own potential, can cope with the normal stresses of life, can work productively and fruitfully, and is able to make a contribution to her or his community” (World Health Organization, 2014). Moreover, well-being is a sign of good mental health (Musschenga, 1997). The concept of well-being stems from the notions of hedonism and eudaimonism in Greek philosophy (Ryan and Deci, 2001). Hedonism is a way of life wherein an individual feels pleasure or happiness and is free from pain and discomfort, whereas eudaimonism entails growing toward one’s hopes and goals and achieving said goals (Ryan and Deci, 2001). In the context of research on well-being, hedonism is defined as subjective well-being and eudaimonism as psychological well-being (Diener, 1984; Ryff, 1989). Specifically, subjective well-being includes “experiencing positive affect” and “avowing happiness or life satisfaction.” Psychological well-being includes “social acceptance,” “social actualization,” “social contribution,” “social coherence,” “social integration,” “personal growth,” “purpose in life,” “autonomy,” “environmental mastery,” “self-acceptance,” and “positive relationships with others” (Keyes, 2005). The ideas of well-being are therefore common to the concept of mental health, meaning that well-being is a suitable indicator of mental health in university students who are also required to live autonomously. Therefore, in this study, we use well-being as an outcome variable reflecting mental health.

Mindfulness may be related to first-year university students’ wellness. Mindfulness is defined as “paying attention in a particular way: on purpose, in the present moment, and non-judgmentally.” (Kabat-Zinn, 1994). Dispositional mindfulness consists of a two-dimensional construct that incorporates focus and quality of attention (Rau and Williams, 2016). Additionally, dispositional mindfulness occurs at various levels within a group, regardless of their mindfulness practice (Brown et al., 2007). Promoting dispositional mindfulness increases social contact (Lindsay et al., 2019). Therefore, dispositional mindfulness may be helpful to establish a new social support system, which is one of the main challenges for first-year students. Dispositional mindfulness can therefore be a protective factor that attenuates the negative influence of living alone on mental health. Several studies have reported that dispositional mindfulness can act as a moderator to the relationship between social factors and well-being. For example, the positive relationship between perceived social support and well-being is promoted by dispositional mindfulness (Stallman et al., 2018). Furthermore, dispositional mindfulness can be seen as a moderator for the relationship between income and well-being (Sugiura and Sugiura, 2018). Dispositional mindfulness acts as a moderator for the relationship between social factors and well-being because awareness and observation without making judgments increases the enjoyment of experiences and can highlight the joy in every aspect of an individual’s daily experience (Sugiura and Sugiura, 2018). Therefore, even if an individual’s living environment is not satisfactory, if their dispositional mindfulness is high, it is possible to find pleasure in life, which may lead to the individual taking action toward social contact. Several studies have reported on the effectiveness of interventions, including mindfulness, to manage the well-being of various populations, including university students (Räsänen et al., 2016; Levin et al., 2017; Tomlinson et al., 2018). Additionally, a meta-analysis showed that among cognitive behavioral variables, attention – including dispositional mindfulness – is most strongly correlated with university students’ well-being (Irie et al., 2019). Therefore, it can be said that dispositional mindfulness is related to the well-being of first-year university students, particularly for first-year students who live on their own and need to create new social support systems. It is still not clear, however, which psychosocial factors are related to the well-being of first-year university students. These factors may be different than those affecting older university students. To improve the well-being of first-year university students, it is necessary to examine the correlations of psychosocial factors, including those factors which are specifically relevant for these students.

To accurately measure the relationship between dispositional mindfulness, the living conditions and the well-being of first-year university students, we used the stressors as control variables. The reason for using the stressors as control variables is that stressors not only affect well-being (Zhang and Zheng, 2017), but are also known to vary greatly from person to person, especially for university students (Mandai et al., 2005). In Japan, the stressors experienced by university students range from academic factors to personal value exploration, part-time jobs, and social activities (Shima, 1992). Therefore, because university students have diverse lifestyles, individual differences in stressors are significant. A stressor can be a complex confounding factor, which is why it was used as a control variable in this study.

This study aims to examine the relationship between dispositional mindfulness, the living conditions and the well-being of first-year university students in Japan.

## Materials and Methods

### Ethics Statement

This study was carried out in accordance with the recommendations of the Research Ethics Committee of Hokusho University and the protocol was approved by this committee (ID: 2017-019). All subjects provided written informed consent in accordance with the Declaration of Helsinki.

### Participants

A paper questionnaire was delivered to 375 Japanese first-year university students from April 2018 to September 2018 at three private universities (Hokusho University, Ritsumeikan University, and Kōnan Women’s University) in Japan. None of the universities have on-campus residence halls. Of the 268 responses received, six responses were excluded because of missing data. Therefore, the final sample included 262 Japanese first-year university students (156 females and 106 males; *M*_age_ = 18.77 years, *SD*_age_ = 0.85). Table 1 presents the demographic data.

**TABLE 1:** Participants’ demographic data.

	**Participants (*N* = 262)**
Age (year)	18.77 ± 0.85
**Gender (%)**	
Male	106 (40.5)
Female	156 (59.5)
**Living condition (%)**	
Living at home	125 (47.7)
Living alone on their own	137 (52.3)
MAAS	60.10 ± 12.67
SWEMWBS	23.11 ± 5.36
Daily Life Stressor Scale	30.90 ± 20.04

*MAAS, Mindful Attention Awareness Scale; SWEMWBS, Short Warwick-Edinburgh Mental Well-being Scale.*

### Measurement

#### Demographics

Participants were asked about demographic characteristics, such as age, gender, and living condition (“living at parental home,” “living on their own,” or “other”). Regarding the gender question, participants were instructed to respond by filling in blanks, rather than selecting a gender. In this study, all respondents answered either male or female. Considering living condition, in Japan, a subset of first-year college students have to live alone, as universities are often too far away from home (for example, parental homes in rural areas or smaller cities with no universities). Additionally, residence halls for students are not popular in Japan; therefore, most students rent an apartment by themselves and live alone (Yasuda, 2014). Furthermore, as more than 97% of Japanese university students either live in their parental home or on their own (Yasuda, 2014), we did not include other specific options (such as sharing a room with friends). Although there was an “other” option as a living condition, no one chose this option.

#### Dispositional Mindfulness

The Mindful Attention Awareness Scale (MAAS; Brown and Ryan, 2003) is a 15-item questionnaire designed to measure dispositional mindfulness. We used the Japanese version of MAAS (Fujino et al., 2015). Similar to the original MAAS, participants responded using a six-point Likert scale to indicate the extent to which they agreed with each item. Higher scores indicate higher dispositional mindfulness. The Japanese version of the MAAS shows good convergent validity with the rumination subscale of the Rumination Reflection Questionnaire (Trapnell and Campbell, 1999): *r* = −0.44 (Fujino et al., 2015) and good internal consistency concerning the current sample (α = 0.91).

#### Well-Being

The Short Warwick-Edinburgh Mental Well-being Scale (SWEMWBS; Stewart-Brown et al., 2009) is a seven-item questionnaire designed to measure well-being, including both subjective well-being and psychological well-being. We used the Japanese version of the SWEMWBS (Suganuma et al., 2016). As with the original SWEMWBS, participants responded using a five-point Likert scale to indicate the extent to which they agreed with each item. Higher scores indicate higher well-being. The Japanese version of the SWEMWBS shows good convergent validity with the Satisfaction with Life Scale (Diener et al., 1985): *r* = 0.58 (Suganuma et al., 2016), and good internal consistency concerning the current sample (α = 0.88).

#### Stressor

As stated before, daily stressors are known to have an effect on well-being (e.g., Hill et al., 2018). Therefore, we used daily stressors as control variables. We used the Daily Life Stressor Scale for University Students (Shima, 1992), a 23-item questionnaire that assesses common stressors (existential, interpersonal, academic, and physical) among Japanese university students. Participants responded using a five-point Likert scale to indicate the extent to which they agreed with each item. Higher scores indicate that more stressors were experienced. The Daily Life Stressor Scale for University Students shows good convergent validity with the General Health Questionnaire (Goldberg, 1978): *r* = 0.48–0.61 (Shima, 1992), and good internal consistency concerning the current sample (α = 0.95).

### Procedures

At each university, the questionnaires were distributed to the students in classrooms and completed questionnaires were collected in designated collection boxes. The questionnaire package included a consent form and an information sheet for participants to complete in their own time. We explained that those who did not consent to participation in this study would not be disadvantaged in any way, such as receiving lower grades.

### Statistical Analysis

All statistical analyses in this study were conducted using R version 3.5.2 (R Core Team, 2019). First, descriptive statistics concerning demographic data, mindfulness, and well-being were presented as means and standard deviations (*SD*). Second, a hierarchical multivariate regression analysis was conducted to examine the relationship between mindfulness, the living conditions and well-being. Results were adjusted for age, gender, and the number of stressors as the control variables. Predictor variables were entered in four steps: age, gender, and stressor were entered in step 1; living condition was entered in step 2; dispositional mindfulness was entered in step 3; and the interaction effect of living condition and dispositional mindfulness was entered in step 4. For the interaction effect, we used mean-centered predictor variables.

## Results

**FIGURE 1:**
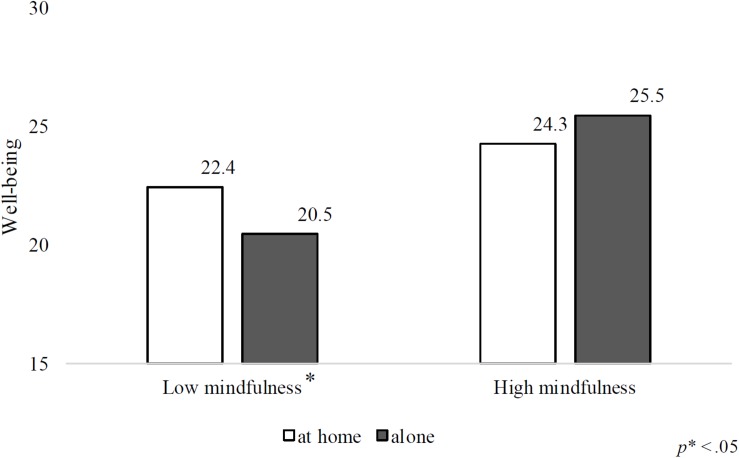
The interaction effect of living condition and mindfulness.

The results of the hierarchical multivariate regression analysis of dispositional mindfulness and living condition on well-being are shown in Table 2. Step 1, which included age, gender, and stressor was significant (*R*^2^ = 0.23, *F*(3,258) = 26.06, *p* < 0.05). Examination of independent variables indicated that number of stressors significantly predicted well-being, but age and gender did not (stressor: β = −0.48, *p* < 0.05, age: β = −0.07, n. s., gender: β = −0.10, n. s.). In step 2, the addition of living condition explained an additional 1% of the variance in predicting students’ well-being (*R*^2^ = 0.24, *F*(4,257) = 19.70, *p* < 0.05), although living condition demonstrated no significant main effect (β = −0.05, n. s.). Step 3, in which dispositional mindfulness was included, explained an additional 7% of the variance in predicting well-being (*R*^2^ = 0.31, *F*(5,256) = 23.28, *p* < 0.05). Dispositional mindfulness showed a significant main effect on well-being (β = 0.45, *p* < 0.05). Step 4, which accounted for the interaction between living condition and dispositional mindfulness, explained an additional 2% of the variance (*R*^2^ = 0.33, *F*(6,255) = 21.26, *p* < 0.05). Furthermore, the interaction between living condition and dispositional mindfulness was significant (β = 0.15, *p* < 0.05). To further investigate the nature of the interaction effect, simple slope analyses were performed separately for dispositional mindfulness at 1 *SD* above and below the mean (Figure 1). These analyses revealed that for students with a dispositional mindfulness score of 1 *SD* below the average (low moderator), there was a significant relationship between living condition and well-being (simple slope = −2.26, *t* = −2.72, *p* < 0.05), indicating that living alone on their own was related to lower well-being. On the other hand, for those with a dispositional mindfulness score of 1 *SD* above the average, the relationship between living condition and well-being was not significant (simple slope = 1.18, *t* = 1.43, n. s.), indicating that higher levels of dispositional mindfulness have a protective effect on the relationship between living condition and well-being. In this regression model, the variance inflation factors were below the standard of 10.0, which indicated that multicollinearity was not a problem in the data.

**TABLE 2:** Results of the hierarchical multivariate regression analysis.

**Steps/Variables**	***F*-value**	***R*^2^**	***b***	***SE***	**β**
*Step 1*	26.06 ^∗^	0.23			
Age			−0.03	0.35	–0.01
Gender (0 = male, 1 = female)			−0.30	0.60	–0.03
Daily Life Stressor Scale			−0.13	0.02	−0.48^∗^
*Step 2*	19.70 ^∗^	0.24			
Age			−0.05	0.35	–0.01
Gender (0 = male, 1 = female)			−0.32	0.60	–0.03
Daily Life Stressor Scale			−0.13	0.02	−0.47^∗^
Living condition (0 = at home, 1 = alone)			−0.49	0.59	–0.05
*Step 3*	23.28 ^∗^	0.31			
Age			−0.02	0.33	–0.00
Gender (0 = male, 1 = female)			−0.20	0.57	–0.02
Daily Life Stressor Scale			−0.10	0.02	−0.34^∗^
Living condition (0 = at home, 1 = alone)			−0.37	0.56	–0.04
MAAS			0.13	0.02	0.31^∗^
*Step 4*	21.26 ^∗^	0.33			
Age			−0.10	0.27	–0.02
Gender (0 = male, 1 = female)			−0.11	0.56	–0.01
Daily Life Stressor Scale			−0.09	0.02	−0.33^∗^
Living condition (0 = at home, 1 = alone)			−0.38	0.55	–0.04
MAAS			0.14	0.02	0.32^∗^
Living condition^∗^MAAS			0.12	0.04	0.15^∗^

*MAAS, Mindful Attention Awareness Scale, ^∗^*p* < 0.05.*

## Discussion

This study examined the relationship between dispositional mindfulness and living condition and well-being among first-year university students in Japan. The results showed a positive relationship between dispositional mindfulness and well-being and a negative relationship between stressors and well-being. Living condition showed no significant relationship to well-being. Therefore, the relationship between dispositional mindfulness and well-being and the relationship between stressors (the control variable) and well-being should have replicated the results of previous studies. Instead, the results revealed a significant relationship between the interaction of living condition and dispositional mindfulness and well-being.

Although several studies have asserted that living condition is one of the most important factors for mental health among first-year university students (e.g., Dyson and Renk, 2006; Sallis et al., 2008), this statement is not based on statistical data. Research has also shown that although students’ living condition does not affect their perceived stress (Saïas et al., 2014), the interaction between students’ living condition and the relationship with their family members affects the perceived stress (Bernier et al., 2005). This infers that the living conditions combined with other specific factors may be related to mental health. Our results showed that dispositional mindfulness and living condition together influences well-being. Specifically, although living on their own was related to lower well-being, a simple slope analysis revealed that higher levels of dispositional mindfulness have a protective effect on the relationship between living condition and well-being. These results suggest that interventions to promote dispositional mindfulness could be an effective strategy to protecting the well-being of first-year university students, especially those who have left their parental homes and live alone. Social support has a positive effect on university students’ mental health (Xu et al., 2018) and promoting dispositional mindfulness reduces loneliness and increases social contact (Lindsay et al., 2019). This indicates that dispositional mindfulness may improve mental health among first-year university students who live alone through other mediating factors.

This study has certain limitations that should be noted. First, we may not have used sufficient control variables, other than stressors. We used the stressors as control variables because they are external factors that could have various effects on the well-being of university students. However, several other factors are known to affect university students’ well-being, such as self-efficacy and coping. Although it is difficult to control all these variables, it is possible that more accurate findings can be obtained by controlling variables that are considered to have a significant influence. Second, the study sample consisted of only Japanese first-year university students. The reason for this specificity of the sample was to examine factors related to transitions that often occur when first entering university. Because of the nature of the sample, the relationship between dispositional mindfulness and living condition and well-being shown in this study may not be generalized to university students in other years. Furthermore, as this research was conducted only in Japan, caution should be used when generalizing the results to countries with cultural backgrounds different from Japan (for example Western or European countries). The results of this study will not necessarily apply to other cultural spheres that are not Asian. Third, we could not verify whether there were participants who had lived alone before entering university. We did not investigate the number of years they had been living alone because in Japan, very few people live alone before entering university. Moreover, there is no data on the proportion of high school students who live alone in Japan. We do know that between 5.4 and 6.6% of those aged from 15 to 19 years old live alone (Statistics Bureau of Japan, 2016); however, as this data includes individuals who are 18 years or older, it could include university students who live alone. Given that the percentage of private university students 18 years or older who are living alone is 35.3% (Japan Student Services Organization, 2018), it is thought that the population of Japanese high school students younger than 18 years living alone is extremely small. In future, to examine the details of the transition to university in Japan and the influence of living condition on the well-being of university students, it will be necessary to collect basic data on participants’ living condition before entering university. Fourth, because of this study’s cross-sectional design, the causal relationship between dispositional mindfulness and well-being of first-year university students could not be clarified. Therefore, it will be necessary to examine, longitudinally, how mental health changes according to the level of dispositional mindfulness of first-year university students.

## Data Availability Statement

The datasets generated for this study are available on request to the corresponding author.

## Ethics Statement

This study was carried out in accordance with the recommendations of the Research Ethics Committee of Hokusho University and the protocol was approved by this committee (ID: 2017-019). All subjects gave written informed consent in accordance with the Declaration of Helsinki.

## Author Contributions

TI and KY carried out data collection. TI drafted the manuscript. KY revised the manuscript.

## Conflict of Interest

The authors declare that the research was conducted in the absence of any commercial or financial relationships that could be construed as a potential conflict of interest.
